# Aconite Poisoning

**Published:** 1882-03

**Authors:** 


### ACONITE POISONING.

Dr. E. T. Reichert (*Philadelphia Medical Times*, November 19, 1881,) gives an analysis of the treatment of forty-one cases of aconite poisoning. Evacuation of the stomach, the administration of large doses of stimulants and the use of external stimuli was the system of treatment pursued in the majority of cases. Opium and its preparations were used in four cases, all of which terminated favorably. In one case five and a half drachms of laudanum were administered in four hours without causing narcotism. Digitalis was administered in two cases in connection with other stimulants. One died and one recovered. The latter who had taken an ounce of Fleming's tincture of aconite, received three hypodermic injections each of twenty minims of tincture of digitalis, within an hour. Amyl nitrite was used with very marked results in one case, and certainly deserves an extended trial in aconite poisoning, as it is a marked cardiac stimulant. Tincture of nux vomica was used in one case with marked benefit to the heart and respiration.—*Chic. Med. Review.*

